# The future of Earth system prediction: Advances in model-data fusion

**DOI:** 10.1126/sciadv.abn3488

**Published:** 2022-04-06

**Authors:** Andrew Gettelman, Alan J. Geer, Richard M. Forbes, Greg R. Carmichael, Graham Feingold, Derek J. Posselt, Graeme L. Stephens, Susan C. van den Heever, Adam C. Varble, Paquita Zuidema

**Affiliations:** 1 National Center for Atmospheric Research, Boulder, CO, USA.; 2 European Centre for Medium-Range Weather Forecasts, Reading, UK.; 3 University of Iowa, Iowa City, IA, USA.; 4 NOAA Chemical Sciences Laboratory, Boulder, CO, USA.; 5 Jet Propulsion Laboratory, California Institute of Technology, Pasadena, CA, USA.; 6 Colorado State University, Fort Collins, CO, USA.; 7 Pacific Northwest National Laboratory, Richland, WA, USA.; 8 Rosenstiel School of Marine and Atmospheric Science, University of Miami, Miami, FL, USA.

## Abstract

Predictions of the Earth system, such as weather forecasts and climate projections, require models informed by observations at many levels. Some methods for integrating models and observations are very systematic and comprehensive (e.g., data assimilation), and some are single purpose and customized (e.g., for model validation). We review current methods and best practices for integrating models and observations. We highlight how future developments can enable advanced heterogeneous observation networks and models to improve predictions of the Earth system (including atmosphere, land surface, oceans, cryosphere, and chemistry) across scales from weather to climate. As the community pushes to develop the next generation of models and data systems, there is a need to take a more holistic, integrated, and coordinated approach to models, observations, and their uncertainties to maximize the benefit for Earth system prediction and impacts on society.

## Teaser

Tighter integration of models and observations of the Earth system should be used to improve weather and climate predictions.

## INTRODUCTION

“It is difficult to make predictions, especially about the future.” The quote has variously been attributed to Niels Bohr (scientist), Yogi Berra (sports star), and Mark Twain (author and humorist). Predictions are one of the main goals of understanding the Earth system on the scale of minutes to centuries and kilometers to the whole globe. The societal value of accurate predictions of the future Earth system is immense and has driven humanity’s need for observing and understanding our world for millennia, since at least Aristotle’s treatise “Meteorology” (Μετεωρολογικά).

In the 21st century, prediction has advanced tremendously. On the basis of observations and theory, we have built models that predict the evolution of weather systems to warn of local extreme events from hours to days in advance, models that predict global weather with lead times up to 2 weeks, models that predict climate anomalies for the next season, and models that project the climate of the next century. Note that there is a difference between prediction and projection. Projections rely on uncertain future boundary conditions such as the level of greenhouse gases in the atmosphere. All these models are constrained and informed by observations of the world around us. This review assesses methods for combining models and observations, including best practices. We show how these methods can be better used to improve predictions.

Skillful weather, air quality, and climate predictions require integration of models with observations. Observations are used to ensure that the starting point for prediction is consistent with the present “state” of the Earth system, as well as for the construction and evaluation of models. The initialization of weather forecasts, using the process of data assimilation (DA) to ingest millions of observations globally each day, has reached a very high degree of sophistication. In contrast, the development and validation of models still relies on approaches that often rely on observations of individual events or small datasets. Inadequacies in modeling detailed processes can lead to persistent errors in our predictions. A better paradigm for bringing together observations and models into an integrated whole would target sources of model error, advance model process representations, and significantly advance our predictive capabilities through increasing our ability to manage growing and diverse data streams.

Also critical is to understand the predictability of the system at any time. Predictability describes the uncertainty in a prediction as a function of both spatial and temporal scales. The rise of ensemble forecasting (using many realizations) has transformed predictability into an envelope of possibilities (or probabilities) rather than a deterministic quantity or single prediction. This matches societal needs for clearly defining prediction uncertainty. Societies design infrastructure for tolerances and extremes, whether for a maximum snow load, minimum stream flow, or minimum or maximum heating/cooling degree days. To optimize systems for this sort of prediction requires a comprehensive, self-consistent, and multilayered fusion of models and observations to characterize uncertainty across many levels.

The level of ambition for Earth system prediction and forecasting is growing, for example, the idea of a digital twin of the Earth (*1*). This requires a blending of models and observations that could be termed model-data fusion. Key questions are whether the models are ready for this: Do models represent complex processes (e.g., deep convective clouds) with sufficient accuracy? Do the observations provide sufficient information to constrain and improve these models? In this review, we explore methodologies to use observations to inform and guide model developments and not just to better initialize predictions.

First, we review current models and observations and their advancement over time (“Models and observations” section). Then, we review the tools used to fuse models and data (“Model-data fusion” section) along with a review of current best practices. Last, in the “Methods for blending observations and models” section, we suggest a path toward taking maximum advantage of the upcoming increases in computing power for modeling and analysis alongside enhanced observations of the Earth system. We summarize this in the “The future of model-data fusion” section.

## MODELS AND OBSERVATIONS

To first order, modeling is limited by advances in computational power. Measurements are limited by technology (e.g., resolution and coverage for a given sensor design) and physical limitations (e.g., information content available in remotely sensed radiation due to varying optical properties of gases, hydrometeors, and aerosols at different wavelengths). Both are limited by a need to balance cost with demonstrable societal benefit. Both models and observations are advancing rapidly. Here, we review the development trajectory of Earth system models and observations to understand the landscape for better bringing together models and observations (both current and future systems).

### Earth system models

It has been nearly 100 years since the concept of numerical weather prediction was first described (*2*). Since the first numerical experiments with electronic computers in the late 1940s, models of weather (*3*) and climate (*4*) have advanced markedly, with improvements in both knowledge and computation power. While the golden age of “Moore’s law” dictating a doubling of computational capability every 18 months may have ended (*5*), new computer architectures and software continue to enable advances in computing power (*6*).

**Fig. 1.**
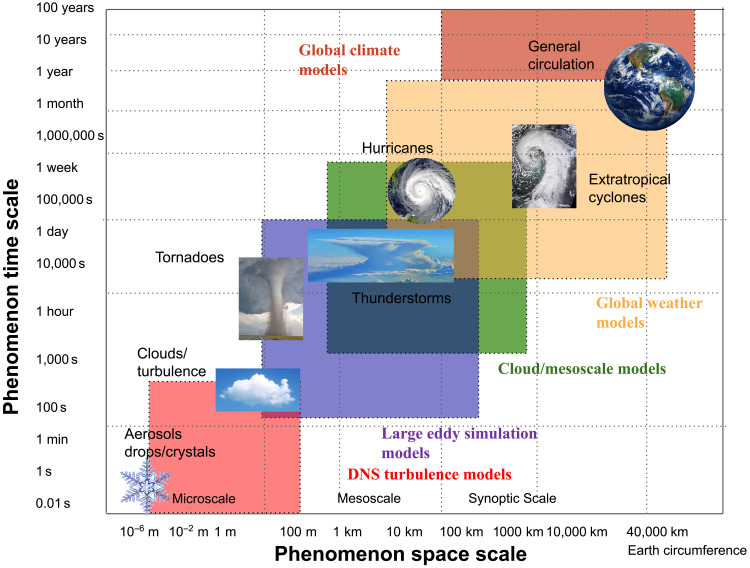
Schematic of atmospheric space and time scales. Schematic representation of space and time scales of atmospheric phenomena from aerosols and cloud drops/crystals at the “microscale” to clouds, tornadoes, and thunderstorms at the “mesoscale” up to tropical cyclones (hurricanes), and extratropical cyclones at the “synoptic scale” up to the general circulation and global climate scale. Types of models and their approximate ranges are colored. The “resolved” scale of a model is typically three to five times the spatial scale, so the effective spatial scale should be shifted to the right. DNS, direct numerical simulation.

Models target specific scales of prediction and focus complexity and computing resources on key processes for a given space and time scale as indicated in Fig. 1. For example, regional mesoscale simulations with 1- to 4-km grid spacing are run for 48 hours or less for severe storm prediction, while global models at coarse (100 km) spatial grids are run for decades to project climate changes. Both of these models often use methods adapted or derived from even more detailed large eddy simulation models (scales of 10 to 100 m). The future focus of prediction extends not only to the state of the system (e.g., temperature, wind speed, rain rate, and runoff volume) but also to applications (e.g., hydrology and air quality), which directly affect human society (*7*). The time scale of prediction is also broadening—e.g., subseasonal to seasonal prediction for agriculture and decadal and century scale for sea-level rise.

In the next decade, models of weather, climate, and air quality will likely evolve to have several characteristics that set them apart from current models. They will be higher resolution, with global weather models reaching a scale of 1 km (*8*) and global climate models reaching 5 to 10 km (*9*). Models will include not only other full components of the Earth system, especially land and chemistry, but also the ocean and cryosphere. Models will also be designed to work across scales of time and space (Fig. 1) from weather to climate. Integrated modeling systems will use common elements for prediction across spatial and temporal scales (*10*). Some current efforts include the Unified Model of the UK Meteorological Office (*11*), as well as plans for the “Destination Earth” program in the European Union (*1*). Integrated Earth System Models will enable targeting of specific locations, facilitated by variable resolution grids that zoom in to areas of interest at resolutions higher than global models (*12*) and can take the place of regional climate models with boundary conditions. Such systems are likely to be designed to provide societally relevant applications such as hydrology or air quality (*13*). Models will also have more complex and varied process representations that function seamlessly across spatial scales. More complex models, with more degrees of freedom, will be harder to constrain. In addition, improvements to models will be more difficult to evaluate, as a change in one Earth system component will affect predictions of the properties of all others. These efforts require conscious coordination across the community, without which there is a risk of fragmentation into specialized models (and observations) that would slow advances in prediction. Last, advances in modeling and predictability will require not only more observations than we have today but also different types of observations, especially those that target fine-scale processes and the interfaces among Earth system components.

### Earth system observations

Weather observations preceded models. Quantitative observations using thermometers go back to 1659 (*14*, *15*). Earth observations from space began with visible images of weather patterns from the first Television Infrared Observation Satellite (TIROS-1) in 1960 (*16*). Global observations range from the Global Telecommunications System (GTS) network of surface stations and radiosondes to numerous individual and “constellations” (coordinated groups) of satellites. In the past 50 years, the availability of data has exploded in several directions, including the number and type of observations. Satellite observations from active and passive sensors measure cloud, aerosol, and environmental properties (*17*) as well as surface properties for the land, ocean, and cryosphere. Commercial aircraft measure winds, temperatures, aerosol, and trace gases (*18*). Robotic subsurface probes measure ocean temperatures, salinity, and currents (*19*). Ground sites and towers measure land surface properties including moisture and snowpack. Remote sensing uses different wavelengths of radiation (e.g., visible, infrared, and microwave) observed from different platforms covering the ocean to outer space. Yet, many key parts of the Earth system remain undersampled. In addition, most current global observing systems observe neither the rapid temporal evolution of the internal structure of weather systems nor the winds that drive them.

Several efforts have charted the future of observing systems (*20*). In the future, observations of the Earth system will have higher spatial and temporal resolution and better precision expanded across additional wavelengths of radiation from multiple perspectives/platforms. An enhanced temporal resolution will be facilitated by closely spaced constellations or swarms of small satellites (*21*), capable of adaptively observing (targeting) features of interest. This could be complemented by enhancements in surface-based remote sensing technology in these target areas. The synergy between passive measurements (e.g., from visible and infrared sensors) and active measurements (e.g., microwave radars and lidars) will be particularly valuable in currently undersampled (remote) regions. The reduced cost of space deployments using smaller sensors like cubesats (*21*) will make observations ubiquitous. Sensors may even leverage smartphone technology (*22*, *23*). Traditionally, only high-value sensors [e.g., aircraft winds and temperature sensors, important for weather forecasting (*24*, *25*)] have been used for prediction. However, many other heterogeneous networks may acquire data useful for weather, air quality, and climate prediction, including household heating and cooling systems, air quality sensors, smartphones, cars (*26*), and even social data like human mobility. These heterogeneous observations vary in quality and calibration. This represents an enormous opportunity and an enormous challenge that requires rethinking how to best integrate diverse and potentially highly uncertain observations with one another, as well as with models.

## MODEL-DATA FUSION

Models and observations are not distinct from one other. Almost all observations have a model built into them; similarly, models are developed with physical laws derived from observations, and evaluated and constrained against observations. Consider the following example. Even a traditional alcohol or mercury thermometer does not measure temperature directly. It measures changes in volume. The thermometer then uses an observation model (the scale on the outside of a thermometer) to convert volume of liquid to a temperature. A thermocouple (electronic thermometer) measures a temperature-dependent change in voltage in a circuit with two metals. The temperature-dependent function is an observation model. A satellite does not directly measure temperature either; likely, it measures voltage on a charge-coupled device (CCD). This voltage is proportional to the intensity of radiation (i.e., radiance) coming from the Earth to the sensor in a particular wavelength range. This radiance is composed of photons that have been emitted from the gases or particles in the Earth’s atmosphere or from the surface of the Earth. The number of photons emitted depends (among other things) on the local temperature. Before reaching the sensor, the photons may have changed their direction of travel many times through scattering from other atmospheric particles and gases, or even from scattering within the surface layer (e.g., in snowpack). To extract geophysical information from the observations, such as the temperature of a layer in the atmosphere, a complex observation model summarizes the complex physical processes involved, from the emission of photons somewhere on the Earth to their conversion to a voltage in the sensor. Given the complexity of processes involved, the observation model likely needs information about the environment that is not available from the sensor itself, but from another measurement or model. For example, the measurement may be sensitive to clouds, surface albedo, or Earth’s surface temperature in addition to the local temperature in an intended measurement volume.

### Components of a model-observation system

**Fig. 2.**
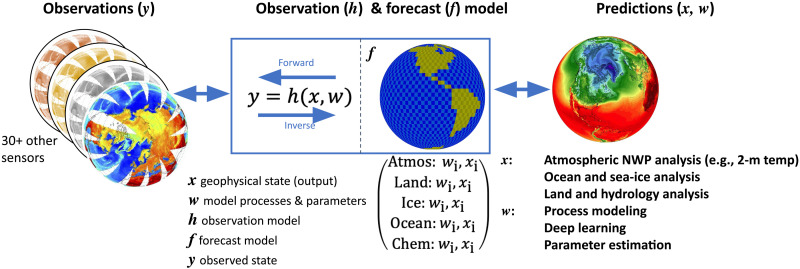
Observations and models to prediction. Schematic of the observation and forecast model linked to the observations (left) and applications or output (right) in an assimilation system. In some systems (4D variational), the forecast model and observation model are tightly coupled and are run forward and backward in time. In other systems, they are more loosely coupled. The goal is to predict the geophysical state (for multiple variables *x_i_*). The system can also be used to improve parameters of the forecast model itself (*w_i_*).

Figure 2 illustrates the key components that link predictive models and observations together in what is commonly called an assimilation system. The observation model (*h*) links a physical quantity of interest (*x*_***i***_) to the internal instrument physical measurement (*y_i_*) using a given set of parameters (*w_i_*). The physical measurement is the intensity of radiation (radiance) in the case of a remote sensing instrument, but the measurement can also be extinction of radiation (e.g., the decrease in radiation that occurs when it is obscured by a cloud), photon counts, or even a change in volume (as for a liquid thermometer). An assimilation system is a way of inverting the observation model (called a forward model) to adjust the physical state (*x_i_*) of the forecast model (*f*) to be closer to the observations (*y*). The forecast model uses the state (*x*) and set of parameters (*w_i_*, some possibly shared with the observation model) with a set of physical equations to generate a prediction. These predictions can be of any desired quantity in the Earth system (*x_i_*), including what we commonly call “weather prediction”: predicting the state of the atmosphere such as winds and temperatures, where *x* = {*u*,*v*,*T*} at some point in the future (here, *u* and *v* represent horizontal wind components and *T* represents temperature). The forecast model itself can be used in different ways. In an assimilation system, the model is run for a short period of time and an observation model is compared directly with observations to bring the initial state closer to the observed state. In a longer-term prediction model, for seasonal or climate time scales, the influence of the initial state fades, and the forecast model parameters (*w_i_*) are critical (*27*). Note that the initial conditions for slowly evolving Earth system components like parts of the land (vegetation), cryosphere (glaciers and ice sheets), and ocean (thermocline and deep ocean) are still important at seasonal or even interannual range, so “short”-term prediction of climate can be defined as a season to a year or even a decade.

### Retrievals

The inversion of the observation model is the key process in inferring the geophysical state *x* from the observations *y*, whether within a DA system or within what is often called a retrieval (*28*). A retrieval is a process that ingests observations from typically one or just a few sources, and produces one geophysical variable (e.g., temperature) at one location or a small subset of locations (e.g., a column in the atmosphere). These retrievals often go on to be treated as “observations” in their own right. To make a retrieval requires knowledge of (or at least an empirical version of) the true observation model. Retrieval of a geophysical variable (*x_i_*) becomes difficult for two reasons: the physical complexity of the observation model and the dependence of the observation model on many imperfectly known geophysical variables that may need to be provided to the retrieval as “a priori” data (unobserved parts of *x*). Observation models for in situ measurements may also need knowledge of the environmental temperature, because it may affect the measurement process. Many observation models for spaceborne measurements need to know properties of the Earth’s surface. For remote sensing observations, the observation (*y*) and externally provided a priori data (this could be represented by *w* in Fig. 2) go into a complex radiative transfer model (*h*; all in Fig. 2) to determine a particular state (*x*) consistent with the inputs. Retrievals often include other types of implicit models like the atmospheric structure of temperature and clouds, or spectral solar insolation. Note how we have just described the generation of an “observation” (*y_i_*) in the same terms that are used to make a future prediction (a future *x_i_*) with a model.

Because of the different inputs to the observation model, representing many degrees of freedom, the observations rarely (if ever) provide enough information to yield a unique answer for the state of the Earth system at a particular location and time and for a particular variable of interest. In mathematical terms, the problem is ill-posed, or alternatively, there is no direct inverse of the observation model. Ancillary (a priori) data are needed to reduce the number of degrees of freedom. Unfortunately, retrieval uncertainty and degrees of freedom vary by situation, confounding the ability to describe uncertainty in the retrievals. The effect of uncertainty in the a priori data can be accounted for, but only with careful mathematical attention to how the retrieval is used (*29*, *30*). Hence, variable information and degrees of freedom by situation are among the largest contributors to “retrieval error” or uncertainty when less sophisticated (or no) error representations are used. An example is the lack of sensitivity to light rain in retrievals based on precipitation radars (*31*).

Retrievals are advancing with better underlying understanding of physical and optical properties. They are also advancing with the ability to ingest more “a priori” data in unique ways. New empirical methods (for estimating *w*) such as neural networks [a type of machine learning under the umbrella of artificial intelligence (AI)] that relate observed quantities (*y*) to the geophysical state (*x*) measured in the laboratory or obtained with other data are starting to be applied to the retrieval problem (*32*). Neural networks have the advantage of being linear combinations of “neuron” weights, which can be easily inverted to produce a forward model of synthetic observations from a model state, as is performed with an observational simulator (see below). Many of these machine learning approaches provide a ranking of how strongly the individual inputs drive the output response, and this can be used to help understand uncertainty and observation model accuracy requirements.

The future of heterogeneous observing systems described above will require different thinking about retrievals. A more heterogeneous observing system might not only include satellite and routine networks (aircraft, ground-based temperatures, and radiosonde soundings) but also make use of field site and campaign data for assimilation targeted at improving process representation, and in specific regimes. This might require a new generation of observation models for complex and special purpose instruments. New sources of measurements could also include swarms of small satellites with reduced properties (single sensor), limited precision, and/or unknown accuracy, but increased frequency. Here, observational uncertainty rises to the fore and must be dealt with in innovative ways. New opportunities may exist in such systems for cross-calibrating sensors to reduce degrees of freedom. Optimal estimation techniques (in many respects common to retrievals and DA) can weight the different measurements based on various criteria to produce a best estimate of the geophysical variable (*33*).

### Data assimilation

Figure 2 illustrates how DA is like a retrieval that attempts to include all available observations, with the observation model run alongside a sophisticated geophysical prediction model (*f*). This can be run forward and (implicitly) backward to optimize the geophysical state to best represent the observations. While the model is designed to be entirely internally self-consistent (dynamics, thermodynamics, and radiation all work together), it drifts away from reality because of errors in the model and/or errors in the initial state, or other critical model inputs, such as emissions in the case of air quality applications. The drift of the model away from the observed state, along with the contribution of individual parameterizations to this drift, allows the identification of parameterizations that require attention and can be used to improve climate models (*34*). DA is designed to keep the model “on track”—either by making the initial conditions (*x*) more realistic or by modifying the model parameters (*w*). To do this, knowledge of the uncertainty in both the observation and the model is critical to decide statistically how much each should be “trusted” and hence how much to push the model toward the observation(s).

Improvements in DA techniques have been a major contributor to the marked improvements in numerical weather prediction (NWP) in recent decades, allowing much more information to be extracted, even from the same observations. For example, forecasts from current systems for the 1980s performed as part of reanalysis efforts far outperform the operational systems of the time (*35*). Key landmarks in NWP (*36*) include the development and operational adoption of three-dimensional (3D)– and 4D-variational assimilation for the atmosphere in the 1990s, the more recent development of ensemble DA methods, and a number of variational-ensemble hybrids. Variants of these methods are used by all leading global centers for NWP and reanalysis. Further developments that are improving forecasts include more direct use of satellite radiances rather than retrieved products and satellite data, which are affected by clouds and precipitation.

For the initialization of other Earth system components (e.g., land and ocean), a wide variety of techniques are used such as optimal interpolation, simplified ensemble Kalman filters, and variational methods described above. Often the methods used operationally for these components are less complex than those used for the atmosphere, and it is an open question as to whether the variety is justified based on different requirements or data availability, or whether a convergence of approach across components and communities would be beneficial. Certainly, for more extended predictions (e.g., subseasonal to seasonal; initialized decadal), a large part of improvements in predictability must be expected to come from the slower varying components of the Earth system (land, ice, and ocean), motivating further focus on assimilation methods for these components (*37*). These efforts are evolving NWP into what (*3*) call NEWP (numerical environmental and weather prediction).

There are further important questions regarding how best to simultaneously initialize Earth system components. Up to now, it has been common practice to perform DA individually in each component, but various research efforts are investigating different degrees of coupled assimilation. These vary from very weakly coupled assimilation (where the assimilation is still essentially done separately in the different components but using a coupled model background) to strongly coupled assimilation (where observations in one component directly affect the state in another component) (*38*). There are many open avenues and research questions, but there is promise of substantial benefits through a more consistent initialization of multiple components (e.g., avoiding shocks and ensuring balanced coupling) and allowing better exploitation of satellite data that are sensitive to the surface state [e.g., sea surface temperature (SST), sea ice, snow, and/or soil moisture] as well as the atmospheric state. In fact, ignoring physical coupling in the Earth system could be considered another source of error in observations. The a priori data often needed for retrievals in assimilation systems can then (like SST) come from part of the assimilation itself.

There is considerable opportunity for improvements in prediction by using observations and assimilation to structurally improve models. Traditionally, a DA system modifies only the state of the model (*x*). However, recent explorations show how observations can be used to modify the “parameters” of a model (*w*) as well (*39*, *40*). In this paradigm, model parameters that are approximations or uncertain are allowed to vary with observations used to obtain their values. In more recent examples, uncertain model components are allowed to vary in time, and observations are used to estimate their evolution (*41*). There is emerging work using observations to develop model structure through machine learning (*42*, *43*). For applications such as air pollution forecasting, there is an additional use of DA to constrain the emissions from fires, dust, and anthropogenic sources by altering the model inputs to better match observations (*44*). Such methods can also be used to improve climate models by training on past observations with short-term forecasts (*45*, *46*).

Another key advance is in the types of observations used in assimilation systems. Traditionally, these have been related to continuous fields of atmospheric state such as temperature, water vapor, winds, or chemical constituents such as ozone. Only recently have observations related to critical but noncontinuous fields, such as clouds or aerosols, begun to be assimilated (*47*–*49*). Clouds are critical for both weather and climate prediction. The absence of cloud assimilation can have a significant effect on the conditions developing soon after initialization that can substantially change the predicted outcome. Assimilation of observations associated with clouds has lagged because (i) many DA approaches do not easily handle discontinuous fields, (ii) observable cloud quantities (e.g., reflectivity) are not directly related to physically simulated cloud properties (number and mass), and (iii) the structural errors in model cloud representations are large. Advancing (i) and (ii) will be critical for prediction and for reducing structural errors in cloud process representation (iii) that are important uncertainties for weather and climate prediction across scales.

## METHODS FOR BLENDING OBSERVATIONS AND MODELS

We have described how DA systems take in situ observations (e.g., temperature from ground stations) as well as remotely observed satellite or ground-based radiances to optimally adjust the model state to fit the observations, linking the two using sophisticated observation models. The current or “initial” state of the Earth system (atmosphere, land, ocean, and cryosphere) is the primary uncertainty in most predictions for time scales less than a “decorrelation time,” approximately the period for the system to lose memory of its prior state. This time scale might be 1 day for convection, 10 days for weather systems, 1 month for soil moisture, and 1 to 2 years for variability like the El Niño Southern Oscillation, or sea-ice variability. The uncertainty in the model representation of processes may have as large an impact on error as initial conditions at longer lead times (*50*, *51*). This is especially true for cloud and precipitation processes, which have short time scales.

Numerical models for weather and climate are heavily constrained by observations in a myriad of ways beyond just initial condition constraints for assimilation systems shown in Fig. 2. Many model processes that cannot be uniquely described by physical laws at the model grid scale are constrained to observations using an empirical relationship (“parameterization”) between observed quantities and predicted state variables. This can be as simple as a regression built on a collection of data from surface measurement sites or aircraft field campaigns (*52*), or it can be as complex as a neural network built with training data, e.g., for a longwave radiation model (*53*). In the absence of sufficient observations, relatively coarse resolution models often rely on finer-resolution models in their development of parameterizations (*54*), despite possible biases in finer-resolution models or scale mismatches. Thus, in the absence of observations, a potential cascade of errors across models exists. Models attempt to interrupt the cascade by constraining processes and the entire state with the basic laws of thermodynamics (conservation of mass and energy) but are then vulnerable to compensating errors. Observational data are also used to evaluate model performance in the prediction of Earth system processes, particularly for regions and processes not necessarily accounted for in the data used in model construction. However, the availability of observations of the time evolution of key Earth system components (thunderstorms, for example) is limited. Hence, errors in parameterizations are often very difficult to identify.

**Fig. 3.**
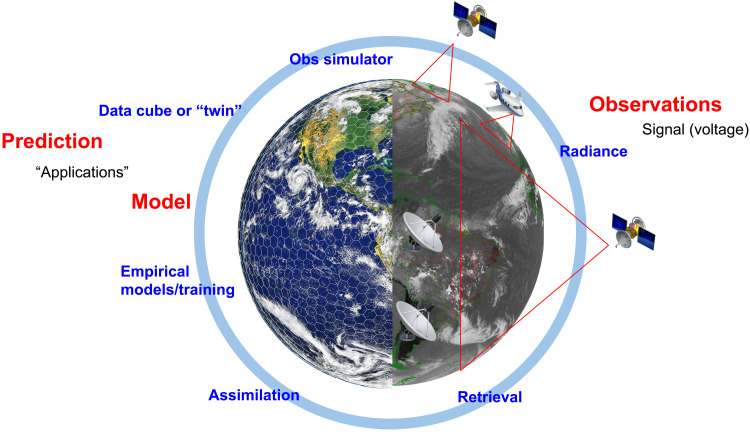
Model-data fusion. Schematic of the fusion of models and observations. The observing system sees only part of the Earth (in space, time, and/or spectral region, noted by the black and white portion of the figure). Satellite radiances (derived from a signal) are observations combined into a retrieval or into an assimilation system. Assimilation systems can be used for training models, and observations can be used to build empirical models as parts of a traditional Earth system model. Such a model produces predictions that are used for applications. The model can be thought of as a “data cube” or digital “twin” of the Earth system, filling out the space, time, and spectral regions not covered by observations. These outputs can be used in an observation simulator to derive synthetic radiances, for either evaluating models or planning observation systems. The ultimate goal is to turn the signal into a prediction for a specific application.

As a result of the integration of models and measurements, it is becoming increasingly difficult to determine where a model stops and observations begin (or vice versa). Figure 3 recasts the model and data system from Fig. 2 in a holistic context. In an assimilation system, the observations are used to inform the prediction by modifying the initial conditions for the next forecast simulation. Observations can also be used to train a model, by minimizing the difference between the model and a set of observations in a global system. For weather prediction models, the error in predictions of past events is used to verify model forecasts. For climate prediction (or projection) models, observations can also be used to evaluate a hierarchy of smaller-scale process models that feed into a global system. Alternatively, the system can be inverted and the model can be used to design observations to minimize prediction uncertainty. Rather than asking “What is the uncertainty in the prediction for a given set of observations?” such an inversion would ask “What observations are needed for a desired prediction uncertainty?” We already perform some targeted observations in this way, e.g., targeted dropsonde observations of tropical cyclones to reduce forecast errors in landfalling storms. Further discussion of targeted observations is in the “Modifying the observing system using models” section.

In this vision of model-data fusion, the model is a self-consistent representation of all the data used to develop, initialize, and evaluate the model. However, the model provides a much more complete multidimensional (3D + time) view of system properties and processes lacking in observations. Viewed another way, a model can be thought of as a dynamic interpolator that fills the gaps between observations that are limited to select variables and often sparse in space and time, using physical knowledge of the transformation processes in the Earth system. For example, in the context of radiance, if the observations provide a black and white view in one wavelength (as illustrated in Fig. 3), the model can provide many additional wavelengths (illustrated by the colors in Fig. 3) and variables consistent with that picture.

### Model evaluation and development

Model development and evaluation now often use data methods that include uncertainty estimates not unlike DA, such as Bayesian evaluation of parameters *w_i_* (*55*) or model emulators constrained by data (*56*, *57*) to optimize model parameters. Recently, the empirical parameterization approach (fitting process rates to data) has taken a jump forward with application of neural networks (*58*–*60*) or Bayesian optimization (*61*). These are essentially higher-order empirical models constrained by (i) observations or (ii) detailed, explicit model solutions that can reproduce observations. Machine learning methods can suffer from typical empirical model problems of overfitting and extrapolation (*60*) and do not necessarily define causal relationships, but recent research is attempting to build in physical laws and constraints (*62*).

Assimilation systems can also be used to help understand model failures in an integrated way. The “analysis increments” that are applied to the model by the assimilation system to nudge it toward observations have structure, which reveals model biases by location and regime. This can be a potent tool for improving models (*63*, *64*).

These efforts make explicit the link between models and observations. Assimilation systems also provide rigorous and objective frameworks for connecting models and observations, with explicit application of tools to evaluate uncertainty. Assimilation can relate both initial condition and model uncertainty to observations. Both initial conditions used for assimilation and the model structure are dependent on data, and hence dependent on the uncertainty in observations and retrievals. This highlights the critical importance of understanding and quantifying observational uncertainty. Future forecasts will have a more holistic approach to probability and uncertainty building on current ensemble forecasts. Probabilistic forecasts should reflect uncertainties in a model and observations that will not be constant in space, in time, or by weather regime.

### Data cubes and digital twins

The goal of model-data fusion is a flexible system within which many questions can be asked, either about the past and how the system works (analysis) or about the future (prediction). A complete “analysis” of the state of the Earth system at some point in the past and the ability to probe that past in many different ways is a “data cube” (*65*) that aligns different databases of observations and models in space and time, representing physical constraints of the Earth system. From a modeling perspective, models can be thought of as a “digital twin,” an engineering term where a computer simulation of a design is created and can also be tested, evaluated, and probed so that the behavior can be predicted (e.g., as in an aircraft design that can be “flown” in a fluid dynamics model to examine performance, stresses, etc.). The design can then be changed before any physical object is built. A digital twin can also be a model for Earth science applications (*1*). The model-data fusion system makes both of these systems possible: analysis of the state of the system at any time and predictions about the future of the system. A model-data fusion system uses both simulations and observations to improve the system over time, working forward and backward between the two as the data cube is used to build, evaluate, and evolve the digital twin.

### Observation simulators

One of the goals of data cubes or digital twins is using the model to directly simulate observations to enable better model evaluation and even determine uncertainty in observations. This can be done with observation simulators, which use the forward observation model to take a model state (*x_i_* in Fig. 2) and produce a synthetic observation (*y_i_*) so that the model can be evaluated against observations with minimal retrieval uncertainties. For example, if the model scene is all ice, or all liquid, what would the observation look like? The same methods can be used in the model. Examples that have been heavily used are radar simulators that include polarimetry and velocity (*66*–*68*) and satellite simulators that include infrared and passive microwave (*69*–*71*). These simulators support model evaluations targeting specific processes, particularly microphysical processes, that produce “fingerprints” in observations (*72*) that can be used to better understand and constrain processes and properties (*73*, *55*).

### Modifying the observing system using models

One further advance is not only using observations to improve models but also using models to improve observations and observing systems. While the development of DA can extract more value from observations, modern assimilation systems can also inform the optimal design of an observational system. Such methods can reveal the most important observations of a given type including optimizing the location and timing of measurements to reduce forecast errors. There are already frameworks to understand the value and importance of specific measurements, such as observation system simulation experiments (OSSEs) (*74*, *75*) and the ensemble data assimilation (EDA) framework for measuring the change in analysis uncertainty as the observing system varies (*76*). These assessments need not be static but can be agile to react to a specific situation, particularly when there is high societal impact. Perhaps the most common example noted is the use of targeted observations in weather forecasting [e.g., “hurricane hunter” aircraft that take measurements to improve forecasts of tropical cyclones, or targeted dropsondes for extratropical cyclones (*77*)]. The impact of targeted observations so far has been limited (*78*), but the broader concept of defining the observing system to maximize societal impact is still important. Models can be used “in reverse” to systematically (statistically) explore how a proposed observation or observing system will affect predictions and their uncertainty. While care is needed in interpretation because observational needs cannot be entirely decoupled from the quality of DA and modeling systems, methods such as OSSEs and EDA close the circle in Fig. 3 through the use of models for improvement of observations used for prediction.

### Uncertainty

Woven throughout the discussion of the above elements is the multilevel treatment of uncertainty, already mentioned 26 times in this review. To be used in a retrieval, observational uncertainty must be defined, with errors specific to each observation method, sensor, and situation (observation errors depend on the physical situation). Knowledge of the observation error correlations in space and time is also important. The retrieval has uncertainty from a priori data and uncertain physics or radiative transfer calculations. Models have uncertainty in their formulation and parameters. For assimilation techniques to work, they need good uncertainty quantification for both the model and the observation to determine how much (as well as where and when) to adjust the model toward a given observation.

Ensemble prediction recognizes this uncertainty and tries to capture it with the goal of quantifying the predictability of a given situation. This is currently done with ensembles by perturbing initial conditions, applying stochastic perturbations to parameterized tendencies (*79*), or stochastically modifying model parameters (*41*) to reflect uncertainties. Ensembles are also now common in climate prediction with initial condition (*80*), multimodel (*81*), and parameter estimation ensembles (*56*). An improved model-data fusion system will enable better understanding and treatment of uncertainty.

## THE FUTURE OF MODEL-DATA FUSION

To chart a path forward, we might ask “What limits our ability to predict?” Is it the quality of models or the uncertainty/lack of observations? At climate time scales, when the effect of most initial conditions fade, limits to prediction are due to errors in model formulations. At short (weather) time scales, a variety of causes create uncertain predictions. For the ocean, prediction may be limited by lack of subsurface observations. For atmospheric turbulence and clouds (and other subgrid processes, see Fig. 1), highly uncertain process formulations at the model grid scale may be the most limiting. Furthermore (and partially as a result of scales), we do not take advantage of all the observations available for assimilation or model development. Despite recent advances, cloud observations remain a prime example of underused observations for assimilation. Thus, both models and observations need to advance, but also the synergy between them via observation model and assimilation techniques. So, the question posed above is a false dichotomy: It is not a binary choice between improving models or observations. Improving specific weather and climate predictions (e.g., precipitation prediction) requires a balanced and interdependent effort between advancing models and advancing observations. The limits to prediction will vary by the quantity and scale (space and time) predicted.

One of the critical challenges in using new observations (of clouds, for example) is the scale mismatch between observations and models. This becomes less of an issue for continuous fields (temperature and wind) as model resolution increases, but it does not help the highly nonlinear problem of assimilation for discontinuous fields like condensed water. Even with stochastic representations of parameters and processes that add dispersion, weather forecast systems have difficulty representing observed variability that results from these nonlinear processes. This may necessitate rethinking DA methods as model resolution and nonlinearities increase. Time scales are an additional underappreciated aspect of assimilation. We can infer changes from continuous station or geosynchronous satellite data, but with limited spatial (ground stations) or spectral (geosynchronous) resolution. Two identical initial states with different time rates of change will produce different futures, so observing and assimilating rates of change, for example, with closely temporally spaced and spatially colocated observations is also important.

To maximally improve predictions, models, observations, and model-data fusion must all improve together. The future of model-data fusion as indicated within the integrated schematic of Fig. 3 consists of several intersecting research areas of unified models, integration of models and observations, and uncertainty characterization within both.

### Unified models

Producing useful predictions across scales of space and time implies the availability of “unified” or integrated modeling systems that can be used for weather, climate, or air quality prediction. Space and time are often linked (Fig. 1): Smaller spatial scale information is often desired to predict extreme events on short lead times (next 6 hours, next 6 days). The same information about extremes is also valuable in a climate context (how will extreme weather events change in the next six decades). A system designed for assimilation can be used to understand and quantify uncertainty relative to observations to inform the climate projection scale. For example, the cloud response to climate change is one of the key uncertainties in climate projection. However, cloud processes occur on fast time scales, so constraining clouds with DA systems that can then also apply to climate is critical (*82*). Constraints from the climate scale, such as global grids and strict requirements on energy and mass conservation, may be important for subseasonal to seasonal forecasting. The key features necessary for each set of scales can be available to both for better predictions at the subseasonal to seasonal scale where both initial states and precise conservation are important.

Existing unified systems are often multiple models or at least nested models, often sharing common components as “loosely connected” systems. The systems and models must be scalable (or configurable), but systems could be simplified if they use the same components in a modular way. For example, a system may not need chemistry for certain applications and should be flexible such that chemistry can be removed or approximated when not needed to reduce computing expense or complexity for purposes of interpretation.

Already in progress are attempts to speed up computationally expensive aspects of models and fill in gaps in development. This includes taking advantage of computing advances and new architectures for large-scale computation such as Graphical Processing Units (GPUs). It also includes using machine learning to speed up parts of models and enable new linearization techniques for adjoint models (*83*) that may speed up development of retrieval algorithms.

A truly comprehensive model-data fusion endeavor would require enormous effort and expertise to build and would have to be a community effort. Is it possible to move toward more coordinated development of either entire systems or common components? It may not be wise to have a single system for all uses (*84*), or practical given the variety of “missions” for each model-data fusion (prediction) system. It is perhaps better to think about building prediction systems in the same way we think about building the observing system: in a modular and interoperable way, sharing ideas and best practices.

Models would benefit from more interoperability, not a single algorithm or set of algorithms, but interoperable standards for building models and linking them to other Earth system components. These standards should include observations and assimilation. There are currently several efforts of this kind in different communities. Examples include the Object Oriented Prediction System (OOPS) for assimilation, the Common Community Physics Package (CCPP) for parameterizations, the National Unified Operational Prediction Capability (NUOPC), and the Earth System Modeling Framework (ESMF) for coupling components.

### Observations integrated into models

Observations need to be thought of as part of the prediction system. As discussed, observations are used at many levels for development and evaluation of models beyond just initializing models. This includes traditional process model development, as well as model evaluation. Existing DA systems have the potential to be used more holistically with integrated modeling systems to bring the weather scale to climate (and vice versa). In turn, models can tell us how to best optimize observations to minimize uncertainty in prediction. Beyond targeted observations for forecasts, there are techniques (EDA and OSSEs) that have the potential to be used more extensively to optimize and design observing systems.

From the perspective of observations, programs should invest in the usability of satellite data. Ideally, the development of simulators and forward operators should occur in tandem with retrieval development and should be considered an integral part of producing satellite data as opposed to an afterthought to facilitate model analysis. A good early example includes the EarthCare satellite and the early preparation of DA frameworks (*48*, *49*, *85*).

When using assimilation, more types of data (e.g., cloud and aerosol observations) should be assimilated. Currently, only a fraction of the information content available from existing observations is being assimilated—those usually related to assimilation of semicontinuous state fields such as winds, temperatures, water vapor, and chemical composition. Advances should be made in assimilation of discontinuous fields including clouds and aerosols. For example, particle number concentration is critical to cloud and radiative processes. These variables currently greatly challenge common DA methods and are not easily observable. Rather, higher-order moments of a cloud particle size distribution are observed (e.g., radar reflectivity is related to the sixth moment of the distribution of spherical particles). These cloud variables could be used to better constrain models.

Sharing observations is one first step. Coordination could be deepened by sharing frameworks for developing assimilation systems and common observation operators for bringing models and data together. The community is already moving in this direction, but such efforts should be strengthened to expand community tools for retrievals and uncertainty analyses of observations. This includes using more extensive radiation frequencies that include active sensors such as radar and lidar.

### Detailed characterization of uncertainty in models and observations

To achieve the goals above, better characterization of models and observations will be necessary. DA methods explicitly require estimates of the observational error. For better predictions, it is not sufficient to just advance observations or models; improved understanding of the uncertainties in observations and models is needed. Integrated model-observation assimilation systems can also produce performance metrics and metrics of bias relative to the observations, which can be used to better characterize model uncertainty.

Satellite systems need more detailed uncertainty estimates from calibration and validation capabilities. Much of the utility of satellite data in DA systems and for model evaluation depends on good error (uncertainty) characterization.

Efforts should also be made to characterize uncertainty and promote development of retrievals for heterogeneous sensors (cubesats, small sats, swarms, and connected devices). These heterogeneous sensor networks will require a combination of fewer, more accurate measurements anchoring more frequent but less accurate data with broader coverage. Statistical methods and machine learning/AI techniques will become very useful for connecting these variable sources of information with variable uncertainty to provide best estimates of the state of the Earth system.

From a modeling perspective, model evaluation should use DA methods to understand parameterization and structural uncertainties and biases. One method for better comparing models to observations is to use satellite simulators to evaluate models in observation space. Another method is to use a full DA assimilation system to improve representation of model parameters (*w_i_*) by tracking and minimizing the increments applied to the model.

Future predictions should have a more holistic approach to probability and uncertainty. There should be better characterization of uncertainty through all aspects of the model-data system, and this uncertainty should be stated as part of the prediction. Implicitly, we do this now with ensembles for both weather and climate. Uncertainty is accounted for explicitly in Bayesian approaches to DA. Integrated systems should use uncertainty characterization to deliver probabilistic predictions (predictions with probabilities, e.g., the chance of an El Niño event or the probability of precipitation). Future systems also need better communication of uncertainty to downstream services and the public.

### Technical advances

Advances in model-data fusion can be enabled by evolving or new technologies and methods in addition to quantitative advances in computation and power and cost reduction of computation (e.g., through accelerators). A qualitative revolution in Earth observations is possible using new sensor and network technologies enabled by consumer electronics such as cubesats or embedded sensors. New observations can be combined with advances in data science, such as machine learning, to advance interpretation of uncertainty in data and causal discovery with large datasets. The potential exists for such methods to improve and automate error characterization, which could result in “data-driven” observation operators and prediction models replacing current empirical treatments with models better constrained by data.

### Summary

Models of the Earth system for weather and climate are advancing rapidly. New and diverse observing systems are coming on-line, adding to the diversity and volume of data. To improve prediction, however, these two aspects of Earth system science need to be better fused. While that “fusion” has been going on since the 1950s with early DA use with NWP (*3*), there is a unique level of fusion now possible with advances in model and observation systems for weather and climate. Observations are not only for initializing forecasts but also can be used to improve models. Models, in turn can be used to provide a more complete digital “twin” of the Earth system for prediction. We have reviewed a variety of current approaches and best practices to show how they can work together. Coordinated investments in models and observing systems could be focused around improving specific important predictions (precipitation and extreme events) to ensure maximum societal benefit. Development of models and observing systems should be guided by prediction limits in a specific area (which may differ for different predictions).

With further coordinated development that better harnesses the resources available to Earth system science, we will build systems critical to improving prediction through multiple, scalable approaches to integrating observations and models. These systems will use customized approaches with shared standards, and common observing systems, with open data access. Community development efforts for shared tools could expand, both for observational systems, as well as the development of observation models (both forward and inverse, i.e., retrievals), and uncertainty tools. Coordination can be facilitated by interoperability of observations and model components. Many of these tools directly relate to the observation-model interface (forward operators and simulators). Development of common observation operators used for retrievals and simulators could be better integrated into observational programs. This would expand the use of data as envisioned here beyond traditional weather applications to a more complete model-data fusion that includes climate and air quality.

This paradigm of better fusion of models and observational data will improve predictive skill. Part of the improvement will come from better uncertainty quantification across Earth system components. Part of the improvement will come from using objective methods to improve models and better observe initial conditions. Such data-model fusion systems will also improve our ability to understand the complex Earth system by facilitating testing of hypotheses about how the Earth system works in previously unavailable ways, providing better societally relevant predictions on scales of hours to decades. This is one prediction for which confidence is high and uncertainty is low.

## References

[1] P. Bauer, B. Stevens, W. Hazeleger, A digital twin of earth for the green transition. Nat. Clim. Chang. 11, 80–83 (2021).

[2] L. F. Richardson, *Weather Prediction by Numerical Process* (Cambridge Univ. Press, 1922).

[3] S. G. Benjamin, J. M. Brown, G. Brunet, P. Lynch, K. Saito, T. W. Schlatter, 100 years of progress in forecasting and NWP applications. Meteorol. Monogr. 59, 13.1–13.67 (2018).

[4] D. A. Randall, C. M. Bitz, G. Danabasoglu, A. S. Denning, P. R. Gent, A. Gettelman, S. M. Griffies, P. Lynch, H. Morrison, R. Pincus, J. Thuburng, 100 years of earth system model development. Meteorol. Monogr. 59, 12.1–12.66 (2019).

[5] M. M. Waldrop, The chips are down for Moore’s law. Nat. News 530, 144–147 (2016). 10.1038/530144a 26863965

[6] C. Edwards, Moore’s law: What comes next? Commun. ACM 64, 12–14 (2021).

[7] B. Beckage, L. J. Gross, K. Lacasse, E. Carr, S. S. Metcalf, J. M. Winter, P. D. Howe, N. Fefferman, T. Franck, A. Zia, A. Kinzig, F. M. Hoffman, Linking models of human behaviour and climate alters projected climate change. Nat. Clim. Chang. 8, 79–84 (2018).

[8] P. D. Dueben, N. Wedi, S. Saarinen, C. Zeman, Global simulations of the atmosphere at 1.45 km grid-spacing with the integrated forecasting system. J. Meteorol. Soc. Jpn. 98, 551–572 (2020).

[9] B. Stevens, M. Satoh, L. Auger, J. Biercamp, C. S. Bretherton, X. Chen, P. Düben, F. Judt, M. Khairoutdinov, D. Klocke, C. Kodama, L. Kornblueh, S.-J. Lin, P. Neumann, W. M. Putman, N. Röber, R. Shibuya, B. Vanniere, P. L. Vidale, N. Wedi, L. Zhou, DYAMOND: The dynamics of the atmospheric general circulation modeled on non-hydrostatic domains. Prog. Earth Planet. Sci. 6, 61 (2019).

[10] G. Brunet, S. Jones, P. M. Ruti, *Seamless Prediction of the Earth System: From Minutes to Months* (World Meteorological Organization, 2015).

[11] M. J. P. Cullen, The unified forecast/climate model. Meteorol. Mag. 122, 81–94 (1993).

[12] X. Huang, A. M. Rhoades, P. A. Ullrich, C. M. Zarzycki, An evaluation of the variable-resolution CESM for modeling California’s climate. J. Adv. Model. Earth Syst. 8, 345–369 (2016).

[13] J. Thépaut, D. Dee, R. Engelen, B. Pinty, The Copernicus programme and its climate change service, in *IGARSS 2018—2018 IEEE International Geoscience and Remote Sensing Symposium* (IEEE, 2018), pp. 1591–1593.

[14] G. Manley, The mean temperature of Central England, 1698–1952. Q. J. R. Meteorol. Soc. 79, 242–261 (1953).

[15] D. E. Parker, T. P. Legg, C. K. Folland, A new daily central england temperature series, 1772–1991. Int. J. Climatol. 12, 317–342 (1992).

[16] A. J. Tatem, S. J. Goetz, S. I. Hay, Fifty years of Earth-observation satellites. Am. Sci. 96, 390–398 (2008). 19498953 10.1511/2008.74.390 PMC2690060

[17] K. Bessho, K. Date, M. Hayashi, A. Ikeda, T. Imai, H. Inoue, Y. Kumagai, T. Miyakawa, H. Murata, T. Ohno, A. Okuyama, R. Oyama, Y. Sasaki, Y. Shimazu, K. Shimoji, Y. Sumida, M. Suzuki, H. Taniguchi, H. Tsuchiyama, D. Uesawa, H. Yokota, R. Yoshida, An introduction to Himawari-8/9—Japan’s new-generation geostationary meteorological satellites. J. Meteorol. Soc. Jpn. 94, 151–183 (2016).

[18] A. Zahn, E. Christner, P. F. J. van Velthoven, A. Rauthe-Schöch, C. A. Brenninkmeijer, Processes controlling water vapor in the upper troposphere/lowermost stratosphere: An analysis of 8 years of monthly measurements by the IAGOS-CARIBIC Observatory. J. Geophys. Res. Atmos. 119, 11,505–11,525 (2014).

[19] D. Roemmich, G. C. Johnson, S. C. Riser, R. E. Davis, J. Gilson, W. Brechner Owens, S. L. Garzoli, C. Schmid, M. Ignaszewski, The Argo program: Observing the global ocean with profiling floats. Oceanography 22, 34–43 (2009).

[20] National Academies of Sciences, Engineering, and Medicine, *Thriving on Our Changing Planet: A Decadal Strategy for Earth Observation from Space* (The National Academies Press, 2018).

[21] G. Stephens, A. Freeman, E. Richard, P. Pilewskie, P. Larkin, C. Chew, S. Tanelli, S. Brown, D. Posselt, E. Peral, The emerging technological revolution in Earth observations. Bull. Amer. Meteor. Soc. 101, E274–E285 (2020).

[22] A. Overeem, H. Leijnse, R. Uijlenhoet, Country-wide rainfall maps from cellular communication networks. Proc. Natl. Acad. Sci. U.S.A. 110, 2741–2745 (2013). 23382210 10.1073/pnas.1217961110 PMC3581927

[23] T. Cao, J. E. Thompson, Remote sensing of atmospheric optical depth using a smartphone sun photometer. PLOS ONE 9, e84119 (2014). 24416199 10.1371/journal.pone.0084119 PMC3885532

[24] R. A. Petersen, On the impact and benefits of AMDAR observations in operational forecasting—Part I: A review of the impact of automated aircraft wind and temperature reports. Bull. Am. Meteorol. Soc. 97, 585–602 (2016).

[25] E. P. James, S. G. Benjamin, B. D. Jamison, Commercial-aircraft-based observations for NWP: Global coverage, data impacts, and COVID-19. J. Appl. Meteorol. Climatol. 59, 1809–1825 (2020).

[26] M. Bartos, H. Park, T. Zhou, B. Kerkez, R. Vasudevan, Windshield wipers on connected vehicles produce high-accuracy rainfall maps. Sci. Rep. 9, 170 (2019). 30655552 10.1038/s41598-018-36282-7 PMC6336807

[27] E. Hawkins, R. Sutton, The potential to narrow uncertainty in regional climate predictions. Bull. Am. Meteorol. Soc. 90, 1095–1108 (2009).

[28] M. Maahn, D. D. Turner, U. Löhnert, D. J. Posselt, K. Ebell, G. G. Mace, J. M. Comstock, Optimal estimation retrievals and their uncertainties: What every atmospheric scientist should know. Bull. Am. Meteorol. Soc. 101, E1512–E1523 (2020).

[29] J. Joiner, A. M. da Silva, Efficient methods to assimilate remotely sensed data based on information content. Q. J. R. Meteorol. Soc. 124, 1669–1694 (1998).

[30] S. Migliorini, On the equivalence between radiance and retrieval assimilation. Mon. Weather Rev. 140, 258–265 (2012).

[31] A. Behrangi, M. Lebsock, S. Wong, B. Lambrigtsen, On the quantification of oceanic rainfall using spaceborne sensors. J. Geophys. Res. Atmos. 117, 20105 (2012).

[32] M. Min, J. Li, F. Wang, Z. Liu, W. P. Menzel, Retrieval of cloud top properties from advanced geostationary satellite imager measurements based on machine learning algorithms. Remote Sens. Environ. 239, 111616 (2020).

[33] G. Feingold, R. Furrer, P. Pilewskie, L. A. Remer, Q. Min, H. Jonsson, Aerosol indirect effect studies at Southern Great Plains during the May 2003 Intensive Operations Period. J. Geophys. Res. Atmos. 111, D05S14 (2006).

[34] T. J. Phillips, G. L. Potter, D. L. Williamson, R. T. Cederwall, J. S. Boyle, M. Fiorino, J. J. Hnilo, J. G. Olson, S. Xie, J. J. Yio, Evaluating parameterizations in general circulation models: Climate simulation meets weather prediction. Bull. Am. Meteorol. Soc. 85, 1903–1916 (2004).

[35] H. Hersbach, B. Bell, P. Berrisford, S. Hirahara, A. Horányi, J. Muñoz-Sabater, J. Nicolas, C. Peubey, R. Radu, D. Schepers, A. Simmons, C. Soci, S. Abdalla, X. Abellan, G. Balsamo, P. Bechtold, G. Biavati, J. Bidlot, M. Bonavita, G. De Chiara, P. Dahlgren, D. Dee, M. Diamantakis, R. Dragani, J. Flemming, R. Forbes, M. Fuentes, A. Geer, L. Haimberger, S. Healy, R. J. Hogan, E. Hólm, M. Janisková, S. Keeley, P. Laloyaux, P. Lopez, C. Lupu, G. Radnoti, P. de Rosnay, I. Rozum, F. Vamborg, S. Villaume, J.-N. Thépaut, The ERA5 global reanalysis. Q. J. R. Meteorol. Soc. 146, 1999–2049 (2020).

[36] R. N. Bannister, A review of operational methods of variational and ensemble-variational data assimilation. Q. J. R. Meteorol. Soc. 143, 607–633 (2017).

[37] S. G. Penny, T. M. Hamill, Coupled data assimilation for integrated earth system analysis and prediction. Bull. Am. Meteorol. Soc. 98, ES169–ES172 (2017).

[38] P. Laloyaux, M. Balmaseda, D. Dee, K. Mogensen, P. Janssen, A coupled data assimilation system for climate reanalysis. Q. J. R. Meteorol. Soc. 142, 65–78 (2016).

[39] S. Kotsuki, Y. Sato, T. Miyoshi, Data assimilation for climate research: Model parameter estimation of large-scale condensation scheme. J. Geophys. Res. Atmos. 125, e2019JD031304 (2020).

[40] A. J. Geer, Physical characteristics of frozen hydrometeors inferred with parameter estimation. Atmos. Meas. Tech. 14, 5369–5395 (2021).

[41] J. Berner, U. Achatz, L. Batté, L. Bengtsson, A. de la Cámara, H. M. Christensen, M. Colangeli, D. R. B. Coleman, D. Crommelin, S. I. Dolaptchiev, C. L. E. Franzke, P. Friederichs, P. Imkeller, H. Järvinen, S. Juricke, V. Kitsios, F. Lott, V. Lucarini, S. Mahajan, T. N. Palmer, C. Penland, M. Sakradzija, J.-S. von Storch, A. Weisheimer, M. Weniger, P. D. Williams, J.-I. Yano, Stochastic parameterization: Toward a new view of weather and climate models. Bull. Am. Meteorol. Soc. 98, 565–588 (2017).

[42] M. Bocquet, J. Brajard, A. Carrassi, L. Bertino, Data assimilation as a learning tool to infer ordinary differential equation representations of dynamical models. Nonlinear Processes Geophys. 26, 143–162 (2019).

[43] A. J. Geer, Learning earth system models from observations: Machine learning or data assimilation? Philos. Trans. R. Soc. A Math. Phys. Eng. Sci. 379, 2020008 (2021). 10.1098/rsta.2020.0089 33583270

[44] N. Huneeus, F. Chevallier, O. Boucher, Estimating aerosol emissions by assimilating observed aerosol optical depth in a global aerosol model. Atmos. Chem. Phys. 12, 4585–4606 (2012).

[45] D. L. Williamson, The evolution of dynamical cores for global atmospheric models. J. Meteorol. Soc. Jpn. 85B, 241–269 (2007).

[46] S. Xie, J. Boyle, S. A. Klein, X. Liu, S. Ghan, Simulations of arctic mixed-phase clouds in forecasts with CAM3 and AM2 for M-PACE. J. Geophys. Res. Atmos. 113, D04211 (2008).

[47] A. J. Geer, K. Lonitz, P. Weston, M. Kazumori, K. Okamoto, Y. Zhu, E. H. Liu, A. Collard, W. Bell, S. Migliorini, P. Chambon, N. Fourrié, M.-J. Kim, C. Köpken-Watts, C. Schraff, All-sky satellite data assimilation at operational weather forecasting centres. Q. J. R. Meteorol. Soc. 144, 1191–1217 (2018).

[48] M. D. Fielding, M. Janisková, Direct 4D-var assimilation of space-borne cloud radar reflectivity and lidar backscatter. Part I: Observation operator and implementation. Q. J. R. Meteorol. Soc. 146, 3877–3899 (2020).

[49] M. Janisková, M. D. Fielding, Direct 4D-var assimilation of space-borne cloud radar and lidar observations. Part II: Impact on analysis and subsequent forecast. Q. J. R. Meteorol. Soc. 146, 3900–3916 (2020).

[50] D. J. Posselt, F. He, J. Bukowski, J. S. Reid, On the relative sensitivity of a tropical deep convective storm to changes in environment and cloud microphysical parameters. J. Atmos. Sci. 76, 1163–1185 (2019).

[51] H. Morrison, M. van Lier-Walqui, A. M. Fridlind, W. W. Grabowski, J. Y. Harrington, C. Hoose, A. Korolev, M. R. Kumjian, J. A. Milbrandt, H. Pawlowska, D. J. Posselt, O. P. Prat, K. J. Reimel, S.-I. Shima, B. van Diedenhoven, L. Xue, Confronting the challenge of modeling cloud and precipitation microphysics. J. Adv. Model. Earth Syst. 12, e2019MS001689 (2020). 10.1029/2019MS001689 PMC7507216 32999700

[52] M. P. Meyers, P. J. DeMott, W. R. Cotton, New primary ice-nucleation parameterizations in an explicit cloud model. J. Applied Met. 31, 708–721 (1992).

[53] V. M. Krasnopolsky, M. S. Fox-Rabinovitz, D. V. Chalikov, New approach to calculation of atmospheric model physics: Accurate and fast neural network emulation of longwave radiation in a climate model. Mon. Weather Rev. 133, 1370–1383 (2005).

[54] T. Schneider, S. Lan, A. Stuart, J. Teixeira, Earth system modeling 2.0: A blueprint for models that learn from observations and targeted high-resolution simulations. Geophys. Res. Lett. 44, 12,396–12,417 (2017).

[55] M. van Lier-Walqui, H. Morrison, M. R. Kumjian, K. J. Reimel, O. P. Prat, S. Lunderman, M. Morzfeld, A Bayesian approach for statistical–physical bulk parameterization of rain microphysics. Part II: Idealized Markov chain Monte Carlo experiments. J. Atmos. Sci. 77, 1043–1064 (2020).

[56] L. A. Regayre, J. S. Johnson, M. Yoshioka, K. J. Pringle, D. M. H. Sexton, B. B. B. Booth, L. A. Lee, N. Bellouin, K. S. Carslaw, Aerosol and physical atmosphere model parameters are both important sources of uncertainty in aerosol ERF. Atmos. Chem. Phys. 18, 9975–10006 (2018).

[57] J. S. Johnson, L. A. Regayre, M. Yoshioka, K. J. Pringle, L. A. Lee, D. M. H. Sexton, J. W. Rostron, B. B. B. Booth, K. S. Carslaw, The importance of comprehensive parameter sampling and multiple observations for robust constraint of aerosol radiative forcing. Atmos. Chem. Phys. 18, 13031–13053 (2018).

[58] S. Rasp, M. S. Pritchard, P. Gentine, Deep learning to represent subgrid processes in climate models. Proc. Natl. Acad. Sci. U.S.A. 115, 9684–9689 (2018). 30190437 10.1073/pnas.1810286115 PMC6166853

[59] N. D. Brenowitz, C. S. Bretherton, Spatially extended tests of a neural network parametrization trained by coarse-graining. J. Adv. Model. Earth Syst. 11, 2728–2744 (2019).

[60] A. Gettelman, D. J. Gagne, C.-C. Chen, M. W. Christensen, Z. J. Lebo, H. Morrison, G. Gantos, Machine learning the warm rain process. J. Adv. Model. Earth Syst. 13, e2020MS002268 (2021).

[61] H. Morrison, M. van Lier-Walqui, M. R. Kumjian, O. P. Prat, A Bayesian approach for statistical–Physical bulk parameterization of rain microphysics. Part I: Scheme description. J. Atmos. Sci. 77, 1019–1041 (2019).

[62] A. McGovern, R. Lagerquist, D. J. Gagne II, G. E. Jergensen, K. L. Elmore, C. R. Homeyer, T. Smith, Making the black box more transparent: Understanding the physical implications of machine learning. Bull. Am. Meteorol. Soc. 100, 2175–2199 (2019).

[63] D. P. Dee, Bias and data assimilation. Q. J. R. Meteorol. Soc. 131, 3323–3343 (2005).

[64] R. Forbes, A. Greer, K. Loniz, M. Ahlgrimm, Reducing systematic errors in cold-air outbreaks. *ECMWF Newsl.* 17–22 (2015).

[65] J. Gray, S. Chaudhuri, A. Bosworth, A. Layman, D. Reichart, M. Venkatrao, F. Pellow, H. Pirahesh, Data cube: A relational aggregation operator generalizing group-by, cross-tab, and sub-totals. Data Min. Knowl. Disc. 1, 29–53 (1997).

[66] B. L. Cheong, R. D. Palmer, M. Xue, A time series weather radar simulator based on high-resolution atmospheric models. J. Atmos. Oceanic Tech. 25, 230–243 (2008).

[67] A. Ryzhkov, M. Pinsky, A. Pokrovsky, A. Khain, Polarimetric radar observation operator for a cloud model with spectral microphysics. J. Appl. Meteorol. Climatol. 50, 873–894 (2011).

[68] M. Oue, A. Tatarevic, P. Kollias, D. Wang, K. Yu, A. M. Vogelmann, The cloud-resolving model radar SIMulator (CR-SIM) Version 3.3: Description and applications of a virtual observatory. Geosci. Model Dev. 13, 1975–1998 (2020).

[69] H. Masunaga, T. Matsui, W. K. Tao, A. Y. Hou, C. D. Kummerow, T. Nakajima, P. Bauer, W. S. Olson, M. Sekiguchi, T. Y. Nakajima, Satellite data simulator unit: A multisensor, multispectral satellite simulator package. Bull. Am. Meteorol. Soc. 91, 1625–1632 (2010).

[70] A. Bodas-Salcedo, M. J. Webb, S. Bony, H. Chepfer, J.-L. Dufresne, S. A. Klein, Y. Zhang, R. Marchand, J. M. Haynes, R. Pincus, V. O. John, COSP: Satellite simulation software for model assessment. Bull. Am. Meteorol. Soc. 92, 1023–1043 (2011).

[71] R. Saunders, J. Hocking, E. Turner, P. Rayer, D. Rundle, P. Brunel, J. Vidot, P. Roquet, M. Matricardi, A. Geer, N. Bormann, C. Lupu, An update on the rttov fast radiative transfer model (currently at version 12). Geosci. Model Dev. 11, 2717–2737 (2018).

[72] A. V. Ryzhkov, J. Snyder, J. T. Carlin, A. Khain, M. Pinsky, What polarimetric weather radars offer to cloud modelers: Forward radar operators and microphysical/thermodynamic retrievals. Atmos. 11, 362 (2020).

[73] K. Suzuki, G. Stephens, A. Bodas-Salcedo, M. Wang, J.-C. Golaz, T. Yokohata, T. Koshiro, Evaluation of the warm rain formation process in global models with satellite observations. J. Atmos. Sci. 72, 3996–4014 (2015).

[74] R. N. Hoffman, R. Atlas, Future observing system simulation experiments. Bull. Am. Meteorol. Soc. 97, 1601–1616 (2016).

[75] X. Zeng, R. Atlas, R. J. Birk, F. H. Carr, M. J. Carrier, L. Cucurull, W. H. Hooke, E. Kalnay, R. Murtugudde, D. J. Posselt, J. L. Russell, D. P. Tyndall, R. A. Weller, F. Zhang, Use of observing system simulation experiments in the United States. Bull. Am. Meteorol. Soc. 101, E1427–E1438 (2020).

[76] F. Harnisch, S. B. Healy, P. Bauer, S. J. English, Scaling of GNSS radio occultation impact with observation number using an ensemble of data assimilations. Mon. Weather Rev. 141, 4395–4413 (2013).

[77] T. M. Hamill, F. Yang, C. Cardinali, S. J. Majumda, Impact of targeted winter storm reconnaissance dropwindsonde data on midlatitude numerical weather predictions. Mon. Weather Rev. 141, 2058–2065 (2013).

[78] S. J. Majumdar, A review of targeted observations. Bull. Am. Meteorol. Soc. 97, 2287–2303 (2016).

[79] R. Buizza, M. Milleer, T. N. Palmer, Stochastic representation of model uncertainties in the ecmwf ensemble prediction system. Q. J. R. Meteorol. Soc. 125, 2887–2908 (1999).

[80] J. E. Kay, C. Deser, A. Phillips, A. Mai, C. Hannay, G. Strand, J. M. Arblaster, S. C. Bates, G. Danabasoglu, J. Edwards, M. Holland, P. Kushner, J.-F. Lamarque, D. Lawrence, K. Lindsay, A. Middleton, E. Munoz, R. Neale, K. Oleson, L. Polvani, M. Vertenstein, The Community Earth System Model (CESM) large ensemble project: A community resource for studying climate change in the presence of internal climate variability. Bull. Am. Meteorol. Soc. 96, 1333–1349 (2014).

[81] T. F. Stocker, D. Qin, G.-K. Plattner, M. Tignor, S.K. Allen, J. Boschung, A. Nauels, Y. Xia, V. Bex, P. M. Midgley, *IPCC, 2013: Climate Change 2013: The Physical Science Basis. Contribution of Working Group I to the Fifth Assessment Report of the Intergovernmental Panel on Climate Change* (Cambridge Univ. Press, 2013).

[82] M. J. Rodwell, T. N. Palmer, Using numerical weather prediction to assess climate models. Q. J. R. Meteorol. Soc. 133, 129–146 (2007).

[83] S. Hatfield, M. Chantry, P. Dueben, P. Lopez, A. Geer, T. Palmer, Building tangent-linear and adjoint models for data assimilation with neural networks. J. Adv. Model. Earth Syst. 13, e2021MS002 521 (2021).

[84] T. Palmer, Climate forecasting: Build high-resolution global climate models. Nature 515, 338–339 (2014). 25409812 10.1038/515338a

[85] R. Voors, D. Donovan, J. Acarreta, M. Eisinger, R. Franco, D. Lajas, R. Moyano, F. Pirondini, J. Ramos, T. Wehr, ECSIM: The simulator framework for EarthCARE, in *Sensors, Systems, and Next-Generation Satellites XI* (International Society for Optics and Photonics, 2007), vol. 6744, p. 67441Y.

